# Supplementary material for: Radiomic biomarkers of locoregional recurrence: prognostic insights from oral cavity squamous cell carcinoma preoperative CT scans
Source: Front Oncol. 2024 Apr 23;14:1380599. doi: 10.3389/fonc.2024.1380599 (PMC11074368; doi:10.3389/fonc.2024.1380599)
Supplement: Supplementary file 1 [file DataSheet_1.docx]

SUPPLEMENTARY INFORMATION:

**Radiomic Biomarkers of Locoregional Recurrence: Prognostic Insights from Oral Cavity Squamous Cell Carcinoma preoperative CT scans**

Xiao et al.

Supplementary Methods:

Radiomic Feature extraction

All IBSI described seven feature classes are extracted using PyRadiomics.

Shape class including: Voxel Volume, Mesh Volume, Surface Area, Sphericity, Surface Volume Ratio, Maximum 2D Diameter (Row), Maximum 2D Diameter (Column), Maximum 3D Diameter, Maximum 2D Diameter (Slice), Major Axis Length, Minor Axis Length, Elongation, Flatness, Least Axis Length.

Firstorder class including: Entropy, Kurtosis, Maximum, Mean, Median, Minimum, Range, Skewness, Uniformity, Variance.

GLCM including: Autocorrelation, Contrast, Cluster Tendency, Correlation, Difference Average, Difference Entropy, Difference Variance, Joint Energy, JointEntropy, Informational Measure of Correlation 1, Inverse Difference Moment, Inverse Difference, Inverse Variance, Maximum Probability, Sum Entropy, Sum Squares.

GLRLM: Short Run Emphasis, Long Run Emphasis, Gray Level Non-Uniformity, Gray Level Non-Uniformity Normalized, Run Length Non-Uniformity, Run Length Non-Uniformity Normalized, Run Percentage, Gray Level Variance, Run Variance, Run Entropy, Low Gray Level Run Emphasis, High Gray Level Run Emphasis, Short Run Low Gray Level Emphasis , Short Run High Gray Level Emphasis , Long Run Low Gray Level Emphasis , Long Run High Gray Level Emphasis .

GLSZM: Small Area Emphasis, Large Area Emphasis , Gray Level Non-Uniformity, Gray Level Non-Uniformity Normalized , Size-Zone Non-Uniformity, Size-Zone Non-Uniformity Normalized, Zone Percentage, Gray Level Variance , Zone Variance, Zone Entropy, Low Gray Level Zone Emphasis, High Gray Level Zone Emphasis , Small Area Low Gray Level Emphasis, Small Area High Gray Level Emphasis , Large Area Low Gray Level Emphasis, Large Area High Gray Level Emphasis.

GLDM: Small Dependence Emphasis, Large Dependence Emphasis , Gray Level Non-Uniformity, Dependence Non-Uniformity , Dependence Non-Uniformity Normalized , Gray Level Variance, Dependence Variance, Dependence Entropy, Low Gray Level Emphasis , High Gray Level Emphasis, Small Dependence Low Gray Level Emphasis, Small Dependence High Gray Level Emphasis, Large Dependence Low Gray Level Emphasis, Large Dependence High Gray Level Emphasis.

NGTDM: Coarseness, Contrast, Busyness, Complexity, Strength.

Aside from the original feature classes, all images are also underwent four filters, wavelet, Laplacian of Gaussian filter Logarithm filter, and Gradient filter. The two risk factors identified in this study are from LoG filtered images. LoG filters are edge enhancement filter, Emphasizes areas of gray level change, where sigma defines how coarse the emphasized texture should be. A low sigma emphasis on fine textures (change over a short distance), where a high sigma value emphasizes coarse textures (gray level change over a large distance).
